# Supplementary material for: Calcium-mediated shaping of naive CD4 T-cell phenotype and function
Source: eLife. 2017 Dec 14;6:e27215. doi: 10.7554/eLife.27215 (PMC5747519; doi:10.7554/eLife.27215)
Supplement: Transparent reporting form [file elife-27215-transrepform.pdf]

eLIFE
1st Floor
24 Hills Road
Cambridge CB2 1JP, UK
P 01223 855340
W elifesciences.org
T @elife

eLife's transparent reporting form

We encourage authors to provide detailed information *within their submission* to facilitate the interpretation and replication of experiments. If you have any questions, please contact us: editorial@elifesciences.org.

Sample-size estimation

- You should state whether an appropriate sample size was computed when the study was being designed
- You should state the statistical method of sample size computation and any required assumptions
- If no explicit power analysis was used, you should describe how you decided what sample (replicate) size (number) to use

Please outline where this information can be found within the submission (e.g., page numbers or figure legends), or explain why this information doesn't apply to your submission:

Sample sizes were chosen to ensure the reproducibility of the experiments and according to the 3Rs of animal ethics regulation.
This statement is included in the Materials and Methods section of this manuscript (in the "Mice" paragraph, page 18)

eLife Sciences Publications, Ltd is a limited liability non-profit non-stock corporation incorporated in the State of Delaware, USA, with company number 5030732, and is registered in the UK with company number FC030576 and branch number BR015634 at the address 1st Floor, 24 Hills Road, Cambridge CB2 1JP | August 2014

Replicates

- You should report how often each experiment was performed
- You should include a definition of biological versus technical replication
- The data obtained should be provided and sufficient information should be provided to indicate the number of independent biological and/or technical replicates
- If you encountered any outliers, you should describe how these were handled
- Criteria for exclusion/inclusion of data should be clearly stated
- High-throughput sequence data should be uploaded before submission, with a private link for reviewers provided (these are available from both GEO and ArrayExpress)

Please outline where this information can be found within the submission (e.g., page numbers or figure legends), or explain why this information doesn't apply to your submission:

Replicate numbers are indicated in each figure legends.
For instance, in figure 1 (pages 28-29):
"(**D**) Proportions of Foxp3$^+$ cells among CD4$^+$ cells are shown as a function of TGFβ1 concentration. Mean ± s.e.m of four independent experiments are shown."

As indicated in the Materials and Methods section of this manuscript (in the "Microarray" paragraph, page 22) "Raw and processed data microarray data are provided in the Gene Expression Omnibus (GEO) under accession number GSE97477." A private link for reviewers is provided here:
https://www.ncbi.nlm.nih.gov/geo/query/acc.cgi?token=mjmbmskijxezpwb&acc=GSE97477

Statistical reporting

- Statistical analysis methods should be described and justified
- Raw data should be presented in figures whenever informative to do so (typically when N per group is less than 10)
- For each experiment, you should identify the statistical tests used, exact values of N, definitions of center, methods of multiple test correction, and dispersion and precision measures (e.g., mean, median, SD, SEM, confidence intervals; and, for the major substantive results, a measure of effect size (e.g., Pearson's r, Cohen's d)
- Report exact p-values wherever possible alongside the summary statistics and 95% confidence intervals. These should be reported for all key questions and not only when the p-value is less than 0.05.

Please outline where this information can be found within the submission (e.g., page numbers or figure legends), or explain why this information doesn't apply to your submission:

Statistical methods used for each experiments are indicated in each figure legends.
For instance, in figure 1 (pages 28-29):
" … . Significance of differences were assessed using a two-tailed paired Student's t-test. Values of $p<0.05$ were considered as statistically significant (** $p<0.01$; ns, not significant).

Our own microarray data were compared with several public Geo Datasets. Correlation analyses were performed using Pearson's correlation test. This information is provided in the figure legends (Figure 3, pages 32-33)

The concentration of TGFβ needed to obtain 50% of the maximal percentage of iTreg cells (Effective Concentration, EC50) was calculated by fitting the dose-response curves of CD4 T_N-cell subsets in the different culture conditions. To this end, the means of 3 to 5 independent experiments were used to build dose response curves using nonlinear least-squares regression to the Hill equation. The model used for this function was $Y=[B+(T–B)] / [1+10^{([LogEC50-X]*HillSlope)}]$, where 'Y' represents $Foxp3^+$ cells as a percentage among $CD4^+$ cells, 'T' and 'B' represent the plateaus at the beginning and end of the curve, respectively, and 'X' represents the concentration of TGFβ added at the beginning of the culture. The absolute EC50 was calculated to interpolate X at 50% with 95% confidence intervals. This information is provided in the "Cell culture and in vitro polarization assays" paragraph of the Materials and Methods section (page 21)

(For large datasets, or papers with a very large number of statistical tests, you may upload a single table file with tests, Ns, etc., with reference to page numbers in the manuscript.)

eLife Sciences Publications, Ltd is a limited liability non-profit non-stock corporation incorporated in the State of Delaware, USA, with company number 5030732, and is registered in the UK with company number FC030576 and branch number BR015634 at the address 1st Floor, 24 Hills Road, Cambridge CB2 1JP | August 2014

1st Floor
24 Hills Road
Cambridge CB2 1JP, UK

P 01223 855340
W elifesciences.org
T @elife

Additional data files ("source data")

- We encourage you to upload relevant additional data files, such as numerical data that are represented as a graph in a figure, or as a summary table
- Where provided, these should be in the most useful format, and they can be uploaded as "Source data" files linked to a main figure or table
- Include model definition files including the full list of parameters used
- Include code used for data analysis (e.g., R, MatLab)
- Avoid stating that data files are "available upon request"

Please indicate the figures or tables for which source data files have been provided:

eLife Sciences Publications, Ltd is a limited liability non-profit non-stock corporation incorporated in the State of Delaware, USA, with company number 5030732, and is registered in the UK with company number FC030576 and branch number BR015634 at the address 1st Floor, 24 Hills Road, Cambridge CB2 1JP | August 2014
